# Prenatal Metformin Therapy Attenuates Hypertension of Developmental Origin in Male Adult Offspring Exposed to Maternal High-Fructose and Post-Weaning High-Fat Diets

**DOI:** 10.3390/ijms19041066

**Published:** 2018-04-03

**Authors:** You-Lin Tain, Kay L. H. Wu, Wei-Chia Lee, Steve Leu, Julie Y. H. Chan

**Affiliations:** 1 Department of Pediatrics, Kaohsiung Chang Gung Memorial Hospital and Chang Gung University College of Medicine, Kaohsiung 833, Taiwan; tainyl@hotmail.com; 2 Institute for Translational Research in Biomedicine, Kaohsiung Chang Gung Memorial Hospital and Chang Gung University College of Medicine, Kaohsiung 833, Taiwan; wlh0701@yahoo.com.tw (K.L.H.W.); leu@mail.cgu.edu.tw (S.L.); 3 Department of Urology, Kaohsiung Chang Gung Memorial Hospital and Chang Gung University College of Medicine, Kaohsiung 833, Taiwan; dinor666@ms32.hinet.net

**Keywords:** developmental origins of health and disease (DOHaD), high-fat, fructose, hypertension, metformin, nitric oxide, nutrient sensing signal, oxidative stress, renin angiotensin system

## Abstract

Widespread consumption of a Western diet, comprised of highly refined carbohydrates and fat, may play a role in the epidemic of hypertension. Hypertension can take origin from early life. Metformin is the preferred treatment for type 2 diabetes. We examined whether prenatal metformin therapy can prevent maternal high-fructose plus post-weaning high-fat diets-induced hypertension of developmental origins via regulation of nutrient sensing signals, uric acid, oxidative stress, and the nitric oxide (NO) pathway. Gestating Sprague–Dawley rats received regular chow (ND) or chow supplemented with 60% fructose diet (HFR) throughout pregnancy and lactation. Male offspring were onto either the ND or high-fat diet (HFA) from weaning to 12 weeks of age. A total of 40 male offspring were assigned to five groups (*n* = 8/group): ND/ND, HFR/ND, ND/HFA, HFR/HFA, and HFR/HFA+metformin. Metformin (500 mg/kg/day) was administered via gastric gavage for three weeks during the pregnancy period. Combined maternal HFR plus post-weaning HFA induced hypertension in male adult offspring, which prenatal metformin therapy prevented. The protective effects of prenatal metformin therapy on HFR/HFA-induced hypertension, including downregulation of the renin-angiotensin system, decrease in uric acid level, and reduction of oxidative stress. Our results highlighted that the programming effects of metformin administered prenatally might be different from those reported in adults, and that deserves further elucidation.

## 1. Introduction

Metformin (1,1-dimethylbiguanide hydrochloride) is the favorite first-line oral blood glucose-lowering treatment for type 2 diabetes [1]. Although metformin shows cardiovascular benefits [2], its antihypertensive effects remain inconclusive [3]. Currently, metformin is used during pregnancy and early childhood for diabetes mellitus [4,5,6]. However, the long-term effects of prenatal metformin exposure on offspring blood pressure (BP) have not been well studied.

Adulthood hypertension can take origin from early-life insults, referred to the developmental origins of health and disease (DOHaD) [7]. The Western diet, which is high in refined sugars and fat content, has been implicated in many diseases, including type 2 diabetes and hypertension. Fructose consumption, mainly from high-fructose (HFR) corn syrup and refined sugars, has grown dramatically in the last half-century [8]. Rats fed HFR diet develop various features of metabolic syndrome, including insulin resistance, obesity, and hypertension [9]. We previously reported that exposure to 60% HFR diet during pregnancy and lactation increased the risk of developing hypertension in adult offspring [10]. On the other hand, high-fat (HFA) diet has been used to generate animal models of hypertension and diabetes [11]. Since postnatal insults can act as a “second hit” to deteriorate earlier programming induced by a first hit, we used to examine whether postnatal HFA (i.e. second hit) can enhance offspring vulnerability to prenatal HFR (i.e., first hit) induced programmed hypertension. Indeed, we found that post-weaning HFA diet exacerbates maternal HFR diet-induced programmed hypertension in adult offspring [12]. Nevertheless, whether prenatal metformin treatment can prevent combined prenatal HFR and post-weaning HFA diets-induced programmed hypertension in adult offspring much remains unknown.

Since BP is regulated by a complex process that includes major contributions from the kidney, and that developing kidney is vulnerable to adverse early-life insults, several mechanisms occurring in the kidney have been proposed to interpret HFR diet-induced renal programming and hypertension. These mechanisms include nutrient sensing signals, uric acid, oxidative stress, and nitric oxide (NO) pathway [12]. Maternal nutritional insults may disturb nutrient sensing signals during pregnancy and lactation periods that leads to hypertension of developmental origin in adult offspring [13]. Nutrient-sensing signaling pathways existing in the kidney include silent information regulator transcript (SIRT), cyclic adenosine monophosphate (AMP)-activated protein kinase (AMPK), peroxisome proliferator-activated receptors (PPARs), PPARγ coactivator-1α (PGC-1α), and mammalian target of rapamycin (mTOR) [14]. Additionally, uric acid has been implicated in the pathogenesis of hypertension as well as HFR-induced type 2 diabetes [15]. The mechanism by which uric acid raises BP might be related to oxidative stress and activation of the renin-angiotensin system (RAS). A number of recent studies support the contribution of oxidative stress involved in programmed hypertension [16,17,18]. Moreover, nitric oxide (NO) controls BP. Imbalance between NO and asymmetric dimethylarginine (ADMA, an endogenous inhibitor of NO synthase) has been linked to the development of hypertension [19,20].

Metformin and ADMA are structural analogs; they have opposite effects at multiple signaling pathways [21]. Additionally, the reported beneficial effects of metformin include AMPK activation and its antioxidant property [1,2,22]. Therefore, we determined whether prenatal metformin therapy can prevent maternal HFR plus post-weaning HFA diets-induced programmed hypertension and also whether the protective effects of metformin are associated with the above-mentioned mechanisms.

## 2. Results

Maternal high-fructose diet had no effect on litter sizes (pups per litter: ND group = 14 ± 0.6; HFR group = 14.3 ± 0.7). HFD and metformin did not affect the survival of male pups (Table 1). The ND/HFA group had a greater body weight (BW) compared with the other 4 groups. The kidney weights were higher in the ND/HFA and HFR/HFA groups compared to ND/ND group. The ratios of kidney weight-to-body weight were higher in the HFR/ND, ND/HFA, and HFR/HFA groups vs. ND/ND group. Prenatal metformin treatment prevented the increases in kidney weight and the ratio of kidney weight-to-body weight. The mean arterial pressures (MAP) of HFR/ND and ND/HFA group were significantly higher than those in ND/ND group. Post-weaning HFA diet caused a significant increase (~15 mmHg) of MAP in HFR/HFA group compared to that in HFR/ND group. This increase in BP was prevented by prenatal metformin therapy. Plasma creatinine level was higher in ND/HFA and HFR/HFA group compared to the other three groups. Uric acid level was higher in HFR/HFA group vs. ND/ND group. The HFR/HFA+Metformin (M) group had the lowest uric acid level among the five groups.

**Figure 1:**
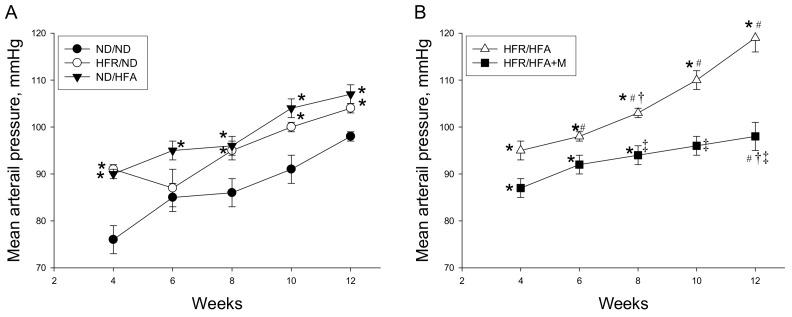
(**A**) Effect of maternal high-fructose (HFR) and post-weaning high-fat (HFA) intake on mean arterial pressure in male offspring; (**B**) Effect of metformin (M) on mean arterial pressure in male offspring exposed to HFR/HFA. * *p* < 0.05 vs. ND/ND, # *p* < 0.05 vs. HFR/ND, † *p* < 0.05 vs. ND/HFA, ‡ *p* < 0.05 vs. HFR/HFA.

As shown in Figure 1, MAP significantly increased in HFR/ND as well as in ND/HFA group compared with that in ND/ND group and was the highest in HFR/HFA group from week 4 through 12. From 8 to 12 weeks of age, MAP was reduced by prenatal metformin therapy in HFR/HFA+M group than that in HFR/HFA group. These data indicated that post-weaning HFA intake aggravated maternal HFR diet-induced programmed hypertension in 12-week-old male offspring, which metformin prevented. Similarly, HFR/HFA increased creatinine and uric acid levels, which were both prevented by prenatal metformin therapy.

**Figure 2:**
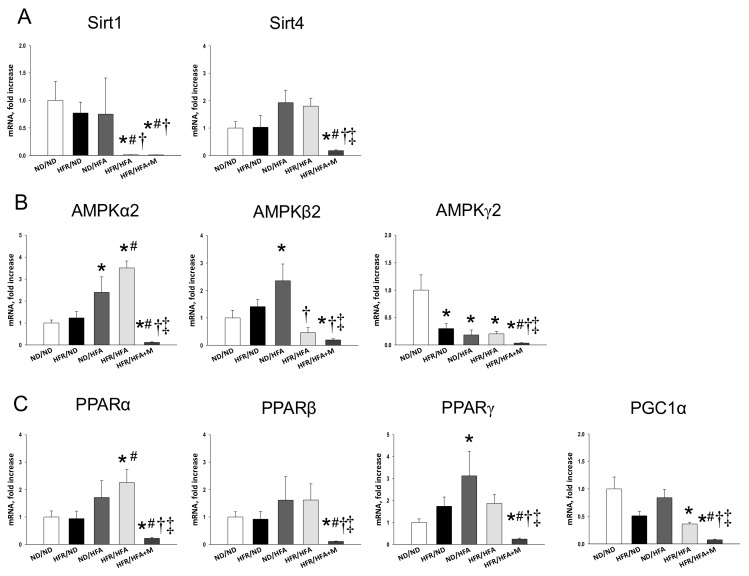
Effect of high-fructose diet (HFR), high-fat (HFA) diet, and metformin (M) on mRNA expression of (**A**) silent information regulator transcript 1 (SIRT1) and 4 (SIRT4); (**B**) AMP-activated protein kinase (AMPK) α-, β- and γ-subunits; and (**C**) Peroxisome proliferator-activated receptor (PPAR) α-, β- and γ-isoforms and PPARγ coactivator-1α (PGC-1α) in 12-week-old male offspring kidneys. * *p* < 0.05 vs. ND/ND, # *p* < 0.05 vs. HFR/ND, † *p* < 0.05 vs. ND/HFA, ‡ *p* < 0.05 vs. HFR/HFA.

**Figure 3:**
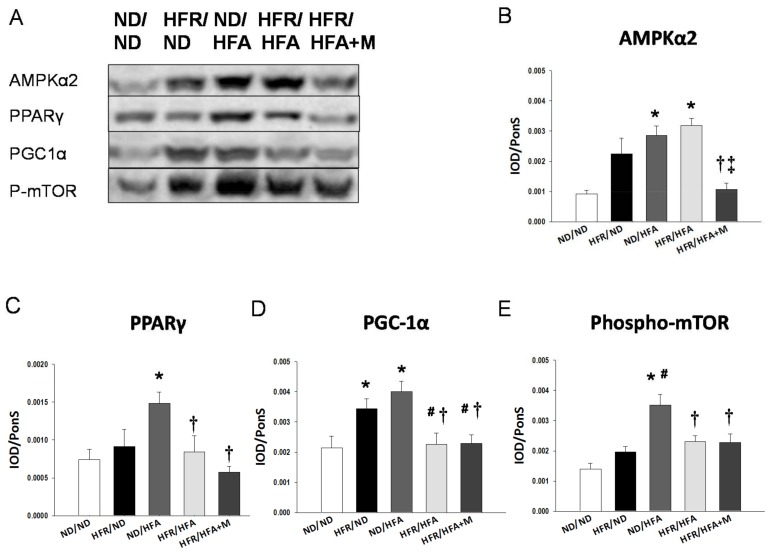
(**A**) Representative western blots and relative abundance of (**B**) AMPKα2 (63 kDa); (**C**) PPARγ (57 kDa); (**D**) PGC-1α (90 kDa); and (**E**) phosphor-mTOR (289 kDa) in male offspring kidneys at 12 weeks of age. * *p* < 0.05 vs. ND/ND, # *p* < 0.05 vs. HFR/ND, † *p* < 0.05 vs. ND/HFA, ‡ *p* < 0.05 vs. HFR/HFA.

We first analyzed genes involved in the nutrient sensing pathway. As shown in Figure 2, renal mRNA expression of *Sirt1*, *Prkag2* (encoding for AMPKγ2)*,* and *Ppargc1a* (encoding for PGC-1α) in HFR/HFA rats were lower, while *Prkaa2* (encoding for AMPKα2) and *Ppara* (encoding for PPARα) were higher than those in ND/ND rats. HFR/HFA+M group had the lowest mRNA expression of *Sirt4*, *Prkaa2*, *Prkag2*, *Ppara*, *Pparb* (encoding for PPARβ), *Pparg* (encoding for PPARγ), and *Ppargc1a* among the five groups. Consistent with the change in mRNA level, Figure 3A showing renal protein level of AMPKα2 was increased in ND/HFA and HFR/HFA group, whereas decreased in the HFR/HFA+M group (Figure 3B) Also, protein level of PPARγ (Figure 3C), PGC-1α (Figure 3D), and phosphorylated mTOR were increased in the ND/HFA group vs. ND/ND group. These changes were restored in HFR/HFA as well as HFR/HFA+M group.

**Figure 4:**
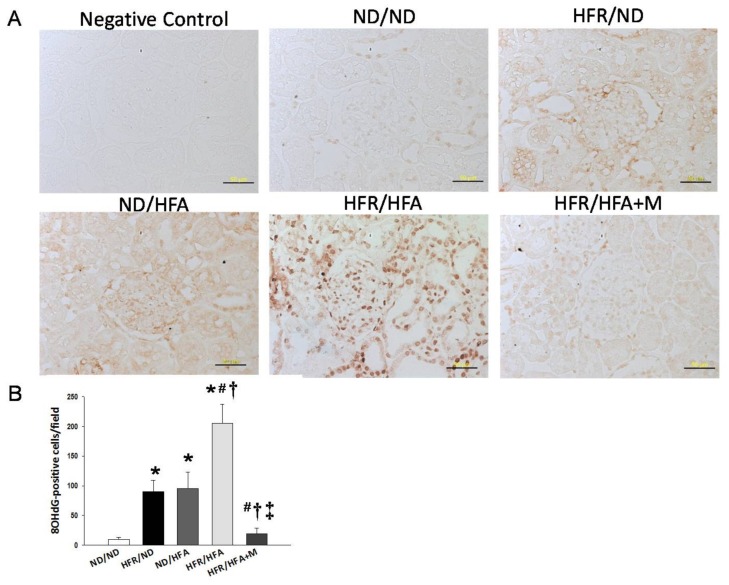
(**A**) Light microscopic findings of 8-hydroxydeoxyguanosine (8-OHdG) immunostaining in the kidney cortex in 12-week-old male offspring. Bar = 50 μm; (**B**) Quantitative analysis of 8-OHdG-positive cells per microscopic field (400×); * *p* < 0.05 vs. ND/ND, # *p* < 0.05 vs. HFR/ND, † *p* < 0.05 vs. ND/HFA, ‡ *p* < 0.05 vs. HFR/HFA.

We next assessed oxidative stress by immunostaining of 8-OHdG. 8-OHdG is a metabolite of oxidative damage to leukocyte DNA, which has been identified as a marker of oxidative stress. Both cytoplasmic and nuclear staining for 8-OHdG in the glomeruli and renal tubules showed little staining in the ND/ND (10 ± 3 positive cells) and HFR/HFA+M (19 ± 10 positive cells) groups, an intermediate level of staining in the HFR/ND (90 ± 19 positive cells) and ND/HFA (95 ± 28 positive cells) groups, and intense staining in the HFR/HFA group (205 ± 32 positive cells) (Figure 4).

Since the interplay between oxidative stress and ADMA-NO pathway involved in the pathogenesis of programmed hypertension [19,20], we hence investigated whether HFR and HFA diets caused the ADMA-NO imbalance and whether metformin can target NO pathway to mediate these alterations (Table 2). Post-weaning high-fat diet caused the decreases in plasma l-arginine levels and l-arginine-to-ADMA ratios in ND/HFA and HFR/HFA rats. HFR/HFA+M group had the lowest plasma l-citrulline and l-arginine levels and l-arginine-to-ADMA ratios among the five groups. In contrast, plasma levels of ADMA and SDMA were highest in HFR/HFA+M group.

**Figure 5:**
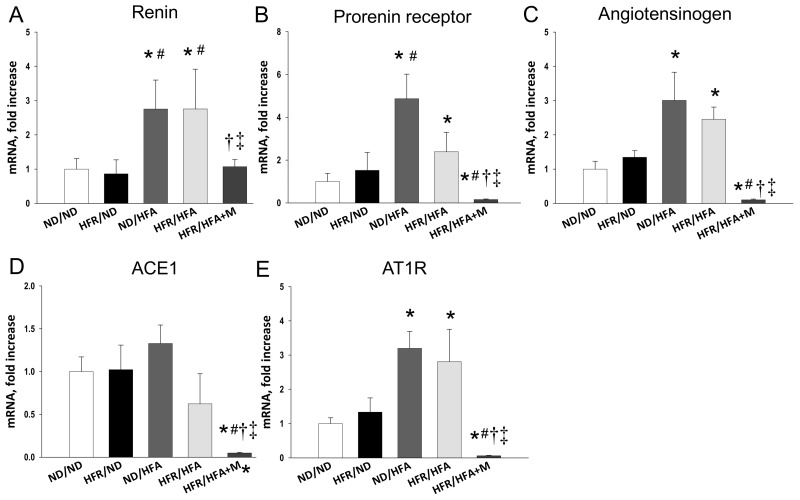
Effect of maternal and post-weaning high-fructose (HFR), post-weaning high-fat (HFA) intake, and metformin (M) on mRNA expression of (**A**) Renin (*Ren*); (**B**) Prorenin receptor (**C**) Angiotensinogen; (**D**) Angiotensin-converting enzyme-1 (ACE1); and (**E**) Angiotensin II type I receptor (AT1R) in male offspring kidneys at 12 weeks of age. * *p* < 0.05 vs. ND/ND, # *p* < 0.05 vs. HFR/ND, † *p* < 0.05 vs. ND/HFA, ‡ *p* < 0.05 vs. HFR/HFA.

We further evaluated the renal mRNA expression of RAS components because they are reported to be involved in inducing hypertension and kidney disease of developmental origin [23,24]. Renal mRNA expression of *Ren*, *Atp6ap2* (encoded for prorenin receptor), *Agt*, and *Agtr1a* (encoded for angiotensin II type I receptor) were higher in ND/HFA group than in those of ND/ND group (Figure 5). However, renal mRNA expression of other RAS components was comparable among offspring in the 4 groups. Similarly, HFR/HFA rats showed significantly increased renal *Ren*, *Atp6ap2*, *Agt*, and *Agtr1a* mRNA expression compared to those in ND/ND group. Moreover, these increased mRNA expressions were reduced by prenatal metformin therapy, which were even lower than those in controls.

## 3. Discussion

Our study provides new insight into the mechanisms by which prenatal metformin therapy protects adult male offspring against hypertension of developmental origin caused by maternal high-fructose plus post-weaning high-fat consumption. The major findings in this study are: (1) combined maternal high-fructose plus post-weaning high-fat diets induced hypertension in adult male offspring, which prenatal metformin therapy prevented; (2) HFR/HFA induced hypertension relates to alterations in the nutrient sensing signals, uric acid, oxidative stress, NO pathway, and RAS; (3) in HFR/HFA-induced programmed hypertension, downregulation of the RAS, decreases in uric acid level, and reduction of oxidative stress were mediated by prenatal metformin, to prevent the development of hypertension.

Despite clear cardiovascular benefits [2], limited data are available regarding rats born from mothers exposed to metformin protecting hypertension of developmental origin. On the other hand, whether metformin can prevent hypertension in adults remains inconclusive [3]. Our two-hit model of the current study is based on the widespread consumption of food containing highly refined carbohydrate and fat by pregnant mothers and their children. Our findings indicate that postnatal HFA intake (i.e. second hit) exacerbates maternal HFR intake (i.e. first hit)-induced programmed hypertension. To the best of our knowledge, the present study is the first to show that prenatal metformin therapy can prevent programmed hypertension of adult male offspring in the HFR/HFA two-hit model. Since that metformin was administered prenatally and that anti-hypertensive effect was starting from eight weeks of age and over time, our findings indicate that the reduction of BP might be due to early intervention with metformin offset the programming process instead of a direct effect. Additionally, maternal high-fructose plus post-weaning high-fat diets caused kidney injury, represented as an elevated creatinine level, which was also prevented by prenatal metformin therapy.

So far, some particular mechanisms contributing to protective effects of metformin have been suggested, such as AMPK activation and its antioxidant property [1,2,22]. We previously found that resveratrol, a known natural activator of AMPK, prevents the combined maternal plus post-weaning high-fat-diets-induced hypertension related to increased protein levels of SIRT1 and AMPKα2 [25]. In contrast, prenatal metformin therapy did not activate but rather inhibited AMPK-related nutrient sensing signals, including AMPKs, SIRT1, SIRT4, PPARs, and PGC-1α, by which it protects adult male offspring against hypertension. Although previous reports showing that metformin protects against hypertension and kidney disease via AMPK activation [26,27], our data implicated that this might not be a major mechanism by which prenatal metformin reprograms the hypertension of developmental origin. Whether prenatal metformin exposure influences the vulnerability of nutrient sensing signals in response to later-life insults to protect against programmed hypertension via AMPK-independent instead of AMPK-dependent mechanisms deserves further clarification.

Another reported protective effect of metformin is mediating NO system [1,2]. Our previous report demonstrated that metformin prevents the development of hypertension in spontaneously hypertensive rats via reducing ADMA and increasing NO production [28]. In this work, we observed that prenatal metformin therapy prevents hypertension related to increased plasma levels of ADMA and SDMA and decreased l-arginine-to-ADMA ratios. Given that both ADMA and SDMA are inhibitors of NO synthase [29], and the l-arginine-to-ADMA ratios represent NO bioavailability, signals formed by NO pathway are supposed to cause vasoconstriction instead of the expected vasodilatation. Thus, our findings suggest that prenatal and postnatal administration of metformin might have opposite effects on nutrient sensing signals and NO pathway, although this remains speculative.

In this work, prevention of programmed hypertension with prenatal metformin therapy in HFR/HFA offspring might be, at least in part, due to blocking the RAS, decreasing uric acid, and reducing oxidative stress. The activation of the RAS, and production of uric acid and oxidative stress, play a key role in the pathogenesis of fructose-induced hypertension [9,15]. First, the RAS is a hormone system that regulates BP and kidney development [30]. Altered kidney RAS components, especially up-regulation of *Ren* at 1 day of age, is relevant to programmed hypertension in a maternal HFR-induced hypertension model [10]. This data are in agreement with our observations showing that HFR/HFA-induced programmed hypertension is associated with the increases of renal mRNA expression of *Ren*, *Atp6ap2*, *Agt*, and *Agtr1*, which promote vasoconstriction and rise BP. Consistent with previous reports showing that cardiovascular protection of metformin is through blockade of the RAS [31], we observed that prenatal metformin therapy restored the increases of RAS components induced by HFR/HFA exposure in the kidney. Second, mounting evidence demonstrates that fructose consumption contributes to uric acid production and high uric acid level is an independent risk factor for hypertension [8,32]. This notion is supported by data presented here showing that HFR/HFA increased plasma uric acid level as well as BP in the adult male offspring. Nevertheless, prenatal metformin therapy concurrently prevented the increases of uric acid level and BP, which was in line with an earlier study showing that metformin reduced blood uric acid level in obese children [33]. Last, oxidative stress is a major mediator in the pathogenesis of hypertension [18,19,20]. We demonstrated the presence of oxidative stress damage, represented by a greater 8-OHdG staining in the kidneys of male offspring exposed to high-fructose and high-fat diets, which was reduced by prenatal metformin therapy. These findings support the theory that alterations of the RAS, uric acid production, and oxidative stress may occur as a result of developmental programming in response to prenatal metformin exposure, priming to permanent changes of these particular pathways, to prevent hypertension of developmental origin.

Our study has a few limitations. First, the anti-hypertensive effects of prenatal metformin therapy may be derived from other organs that control BP. Additional studies are required to examine the effects of prenatal metformin exposure on other organs responsible for BP regulation. Also, the beneficial effects of prenatal metformin therapy in other hypertensive models of developmental origins await further evaluation. Another limitation is that we did not conduct a ND/ND+M group because metformin is currently used during pregnancy with a good safety profile [5]. Nevertheless, the long-term programmed effects of prenatal metformin exposure on normal controls deserve further clarification. Also, the implications of prenatal metformin therapy in the HFR/ND as well as ND/HFA group deserve further clarification. Furthermore, extensive experiments in analyzing protein levels and/or functional activity of the RAS components are required for a thorough examination on the regulation of metformin in the RAS pathway. It is noteworthy that only male offspring were investigated in the current study. Given that metformin has been reported to regulate sex hormones and cause sex-specific effects [34,35], additional studies are required to clarify whether sex-specific differences exist in protective mechanisms of prenatal metformin therapy. Finally, we did not examine other dosing and timing of administration, whether these changes produce same reprogramming effects on HFR/HFA induced hypertension await further evaluation.

## 4. Materials and Methods

### 4.1. Animal Models

The experimental protocol was approved by the Institutional Animal Care and Use Committee of the Kaohsiung Chang Gung Memorial Hospital and are in close agreement with the recommendations of the Guide for the Care and Use of Laboratory Animals of the National Institutes of Health (Protocol No.: 2015120102, 13 January 2016). Virgin Sprague–Dawley (SD) rats (12–16 weeks old) were obtained from BioLASCO Taiwan Co., Ltd. (Taipei, Taiwan). Animals were maintained in an AAALAC-accredited animal facility, housed in a 12-h light-dark cycle. Male SD rats were housed with individual females until mating was confirmed by the examination of a vaginal plug. Pregnant SD rats received regular chow (ND; *n* = 4) or chow supplemented with 60% fructose (HFR; *n* = 8) throughout pregnancy and lactation periods [36]. In order to equal the received quantity of milk and maternal pup care, litters were standardized to eight pups per litter at birth. Because males are prone to develop hypertension occurs at a higher rate and at an earlier age compared to females [37], only male offspring were selected from each litter and used in subsequent experiments. Male offspring were assigned to five groups ((maternal diet/post-weaning diet; *n* = 8 for each group): ND/ND, HFR/ND, ND/HFA, HFR/HFA, and HFR/HFA+M. Male offspring rats were administered either a normal diet with regular chow (ND) or a high-fat hypercaloric diet (HFA; D12331, Research Diets, Inc., New Brunswick, NJ, USA; 58% fat (hydrogenated coconut oil) plus high sucrose (25% carbohydrate)) from weaning to 3 months of age. In addition to HFR/HFA diets, mother rats in the HFR/HFA+M group received metformin 500 mg/kg/day via gastric gavage for 3 weeks during pregnancy period. The dose of metformin used here was based on our previous study conducted with rats [28]. BP was measured in conscious and previously trained offspring by using an indirect tail-cuff method (BP-2000, Visitech Systems, Inc., Apex, NC, USA) at 4, 6, 8, 10, and 12 weeks of age [10]. Three BP measures were taken at 5-min intervals. All offspring were killed at 12 weeks of age. Rats were anesthetized using an intraperitoneal injection of ketamine (50 mg/kg) and xylazine (10 mg/kg), then euthanized by an intraperitoneal overdose of pentobarbital. Heparinized blood samples were collected at the end of the study. The kidneys were subsequently collected and stored at −80 °C for future analysis.

### 4.2. High-Performance Liquid Chromatography (HPLC)

Blood creatinine and uric acid levels were determined by high-performance liquid chromatography (HPLC, HP series 1100; Agilent Technologies Inc., Santa Clara, CA, USA) according to methods described previously [36]. The levels of several components of the NO pathway, including l-citrulline, l-arginine, ADMA, and SDMA, were measured using HPLC with the *O*-phtalaldehyde-3-mercaptoprionic acid derivatization reagent [22]. Standards contained concentrations of 1–100 mM l-citrulline, 1–100 mM l-arginine, 0.5–5 mM ADMA, and 0.5–5 mM SDMA. The recovery rate was around 95%.

### 4.3. Quantitative Real-Time Polymerase Chain Reaction (PCR)

RNA was extracted from the kidney cortex according to previously described methods [10]. Several genes related to the nutrient sensing signaling pathway were analyzed in this study, including sirtuin-1 (*Sirt1*), sirtuin-4 (*Sirt4*), protein kinase, AMP-activated, subunit-α2 (*Prkaa2*), -β2 (*Prkab2*), and -γ2 (*Prkag2*), peroxisome proliferator-activated receptor (PPAR)-α (*Ppara*), -β (*Pparb*), and -γ (*Pparg*), and PPARγ coactivator 1-α (encoded by *Ppargc1a*). Additionally, several components of the RAS analyzed in this study included renin (*Ren*), (pro)renin receptor (*Atp6ap2*), angiotensinogen (Agt), angiotensin converting enzyme-1 (*Ace*), and angiotensin II type I receptor (*Agtr1a*). We used 18S rRNA gene (*Rn18s*) as a reference gene. Primer sequences are provided in Table 3. Comparative threshold (*C*_t_) values were used for all manipulations and were first normalized to average *Rn18s* value by calculating the Δ*C*_t_ for each sample. Values were then calculated relative to control to generate a ΔΔ*C*_t_ value. The fold-increase of the experimental sample relative to the control was calculated using the formula 2^−ΔΔ*C*t^.

### 4.4. Western Blot

Samples were subjected to electrophoresis, western blot, and antibodies icubation using the methods published previously [10]. Briefly, 200 μg of kidney cortex were loaded on a 6–10% polyacrylamide gel and separated by electrophoresis (200 volts, 90 min). The proteins were then electrotransferred to a nitrocellulose membrane (GE Healthcare Bio-Sciences Corp., Piscataway, NJ, USA). The membranes were incubated with Ponceau S red (PonS) stain solution (Sigma-Aldrich, St. Louis, MO, USA) for 10 min on the rocker. After blocking with phosphate-buffered saline-Tween (PBS-T) containing 5% dry milk. The blots were incubated overnight at 4 °C with a 1:1000 dilution of primary antibodies (goat anti-AMPKα2, Santa Cruz Biotechnology, Santa Cruz, CA, USA; rabbit anti-PPARγ, Abcam, Cambridge, MA, USA; rabbit anti-PGC-1α, Santa Cruz Biotechnology; and rabbit anti-phosphorylated mTOR, Cell Signaling, Danvers, MA, USA). Following five washes with 0.1% Tween-Tris-buffered saline (TBS-T), the membranes were incubated for 1 h with horseradish peroxidase-labeled secondary antibody diluted 1:1000 in TBS-T. Bands were visualized using SuperSignal West Pico reagent (Pierce; Rockford, IL, USA) and quantified by densitometry as integrated optical density (IOD), normalized to PonS staining to correct for variations in total protein loading and for an internal standard. The protein abundance was represented as IOD/PonS.

### 4.5. Immunohistochemistry Staining

Paraffin-embedded tissue sectioned at 3-µm thickness was deparaffinized in xylene and rehydrated in a graded ethanol series to phosphate-buffered saline. 8-Hydroxydeoxyguanosine (8-OHdG) is a DNA oxidation product that was measured to assess DNA damage. Following blocking with immunoblock (BIOTnA Biotech., Kaohsiung, Taiwan), the sections were incubated for 2 h at room temperature with an anti-8-hydroxydeoxyguanosine (8-OHdG) antibody (1:100, JaICA, Shizuoka, Japan). Immunoreactivity was revealed using the polymer-horseradish peroxidase (HRP) labelling kit (BIOTnA Biotech) and 3,3’-diaminobenzidine (DAB) as the chromogen. A negative control of identical staining omitting incubation with a primary antibody was used. 8-OHdG-positive cells in high-power fields (400×) in the renal sections were quantitatively analyzed as previously described [12].

### 4.6. Statistical Analysis

Values given in figures and tables represent mean ± standard error of the mean. Statistical significance was determined using one-way ANOVA with Tukey post hoc test for multiple comparisons. Significance of BP data were determined using two-way repeated-measures ANOVA and Tukey post hoc test. In all cases, a *p*-value of 0.05 was accepted as statistical significance. All analyses were performed using the Statistical Package for the Social Sciences software (SPSS, Chicago, IL, USA).

## 5. Conclusions

In conclusion, prenatal metformin therapy reduced BP in adult male offspring rats that was induced by maternal high-fructose and post-weaning high-fat consumption. Some important mechanisms involved in the beneficial action of prenatal metformin therapy on HFR/HFA-treated offspring kidneys, including blockade of the RAS, reduction of oxidative stress, and decreases in uric acid level. Unlike common mechanisms of metformin reported in adults [1,2], our study highlighted that prenatal administration of metformin had different effects on nutrient sensing signals and NO pathway in the adult male offspring. These novel findings compel us to further clarify the programming effects of prenatal metformin exposure in the protection of hypertension to develop a novel intervention for hypertension of developmental origins related to excessive intake of fructose and fat.

## Figures and Tables

**Table 1:** Measures of body and organ weights, blood pressures, and functional parameters in 12-week-old male offspring exposed to maternal high fructose intake, post-weaning high-fat diet, and metformin.

Groups	ND/ND	HFR/ND	ND/HFA	HFR/HFA	HFR/HFA+M
Mortality	0%	0%	0%	0%	0%
BW (g)	413 ± 11	398 ± 10	489 ± 12 ^a,b^	407 ± 13 ^c^	418 ± 15 ^c^
Left kidney weight (g)	1.57 ± 0.1	1.68 ± 0.05	2.02 ± 0.04 ^a,b^	1.7 ± 0.06 ^a^	1.5 ± 0.08 ^c,d^
Left kidney weight/100 g BW	0.38 ± 0.02	0.42 ± 0.01 ^a^	0.41 ± 0.02 ^a^	0.43 ± 0.02 ^a^	0.36 ± 0.01 ^b,c,d^
Liver weight (g)	16.8 ± 1	15.5 ± 0.7	19.2 ± 1	17.2 ± 0.8	15.9 ± 1.3
Liver weight/100 g BW	4.12 ± 0.3	3.9 ± 0.12	3.92 ± 0.16	4.22 ± 0.16	3.78 ± 0.18
Mean arterial pressure (mm Hg)	98 ± 1	104 ± 1 ^a^	107 ± 2 ^a^	119 ± 3 ^a,b,c^	98 ± 3 ^b,c,d^
Creatinine (μmol/L)	16.0 ± 0.8	16.7 ± 0.2	25.5 ± 0.2 ^a.b^	21.9 ± 0.6 ^a,b^	13.6 ± 0.6 ^c,d^
Uric acid	73.5 ± 3.1	86.3 ± 2.7	76.5 ± 2.9	93.3 ± 3.4 ^a^	36.2 ± 1.6 ^a,b,c,d^

HFR/ND, maternal high-fructose intake; ND/HFA, post-weaning high-fat intake; HFR/HFA, maternal high-fructose plus post-weaning high-fat intake; HFR/HFA+M, maternal high-fructose plus post-weaning high-fat intake and treated with metformin. BW, body weight; *n* = 8/group; ^a^ *p* < 0.05 vs. ND/ND; ^b^ *p* < 0.05 vs. HFR/ND; ^c^ *p* < 0.05 vs. ND/HFA; ^d^ *p* < 0.05 vs. HFR/HFA.

**Table 2:** Plasma l-citrulline, l-arginine, ADMA, and SDMA levels in 12-week-old male offspring exposed to high-fructose diet (HFR), high-fat (HFA) diet, and metformin (M).

Groups	ND/ND	HFR/ND	ND/HFA	HFR/HFA	HFR/HFA+M
l-citrulline	57.2 ± 1.1	51.4 ± 1	68.8 ± 1.5	56.6 ± 1.6	107.7 ± 7.8 ^a,b,c,d^
l-arginine	241.1 ± 4.7	208 ± 5.8	167.3 ± 8.5 ^a^	158.9 ± 2.1 ^a^	155 ± 6.6 ^a,b,c,d^
ADMA	1.01 ± 0.03	1.02 ± 0.04	0.93 ± 0.01	0.86 ± 0.01	1.8 ± 0.1 ^a,b,c,d^
SDMA	0.61 ± 0.01	0.58 ± 0.01	0.55 ± 0.01	0.52 ± 0.01	1.1 ± 0.02 ^a,b,c,d^
l-arginine-to-ADMA ratio	233 ± 1	202 ± 3	179 ± 8 ^a^	176 ± 3 ^a^	92 ± 3 ^a,b,c,d^

ADMA, asymmetric dimethylarginine; SDMA, symmetric dimethylarginine; ^a^ *p* < 0.05 vs. ND/ND; ^b^ *p* < 0.05 vs. HFR/ND; ^c^ *p* < 0.05 vs. ND/HFA; ^d^ *p* < 0.05 vs. HFR/HFA.

**Table 3:** Quantitative real-time polymerase chain reaction primers sequences.

Gene	Forward	Reverse
*Sirt1*	5 tggagcaggttgcaggaatcca 3	5 tggcttcatgatggcaagtggc 3
*Sirt4*	5 ccctttggaccatgaaaaga 3	5 cggatgaaatcaatgtgctg 3
*Prkaa2*	5 agctcgcagtggcttatcat 3	5 ggggctgtctgctatgagag3
*Prkab2*	5 cagggccttatggtcaagaa 3	5 cagcgcatagagatggttca 3
*Prkag2*	5 gtgtgggagaagctctgagg 3	5 agaccacacccagaagatgc 3
*Ppara*	5 agaagttgcaggaggggatt 3	5 ttcttgatgacctgcacgag 3
*Pparrb*	5 gatcagcgtgcatgtgttct 3	5 cagcagtccgtctttgttga 3
*Pparg*	5 ctttatggagcctaagtttgagt 3	5 gttgtcttggatgtcctcg 3
*Ppargc1a*	5 cccattgagggctgtgatct 3	5 tcagtgaaatgccggagtca 3
*Ren*	5 aacattaccagggcaactttcact 3	5 acccccttcatggtgatctg 3
*Atp6ap2*	5 gaggcagtgaccctcaacat 3	5 ccctcctcacacaacaaggt 3
*Agt*	5 gcccaggtcgcgatgat 3	5 tgtacaagatgctgagtgaggcaa 3
*Ace*	5 caccggcaaggtctgctt 3	5 cttggcatagtttcgtgaggaa 3
*Agtr1a*	5 gctgggcaacgagtttgtct 3	5 cagtccttcagctggatcttca 3
*Rn18s*	5 gccgcggtaattccagctcca 3	5 cccgcccgctcccaagatc 3

## Abbreviations

ADMA	Asymmetric dimethylarginine
AMPK	AMP-activated protein kinase (AMPK)
AT1R	Angiotensin II type I receptor
DOHaD	Developmental origins of health and disease
HFA	High-fat diet
HFR	High-fructose diet
mTOR	Mammalian target of rapamycin
ND	Normal diet
PGC-1α	PPARγ coactivator-1α
PPAR	Peroxisome proliferator-activated receptor
RAS	Renin-angiotensin system
SDMA	Symmetric dimethylarginine
SIRT1	Sirtuin-1
SIRT4	Sirtuin-4
